# High‐Definition and Autofluorescence Bronchoscopic Imaging for Evaluating Epithelial Changes in Squamous Cell Lung Cancer After Neoadjuvant Immunochemotherapy: A Case Report

**DOI:** 10.1111/1759-7714.70096

**Published:** 2025-05-21

**Authors:** Kei Morikawa, Koji Kojima, Hideki Marushima, Yoshiya Sugiura, Junki Koike, Hisashi Saji, Masamichi Mineshita

**Affiliations:** ^1^ Department of Respiratory Diseases St. Marianna University School of Medicine Kawasaki Japan; ^2^ Department of Chest Surgery St. Marianna University School of Medicine Kawasaki Japan; ^3^ Department of Pathology St. Marianna University School of Medicine Kawasaki Japan

**Keywords:** autofluorescence imaging, high‐definition bronchoscope, neoadjuvant chemotherapy

## Abstract

In recent years, perioperative immune checkpoint inhibitors have become indicated for early‐stage lung cancer, emphasizing the importance of high‐resolution endoscopic evaluation of preoperative drug therapy. At the initial evaluation, a male patient in his 60s presented with a primary lesion obstructing the right upper lobe bronchus. After three courses of neoadjuvant immunochemotherapy, chest computed tomography and endoscopic examinations showed a near‐complete response. Narrow‐band imaging indicated that subepithelial vascular regularity and distribution patterns were within normal limits. However, autofluorescence imaging (AFI) revealed a magenta‐colored area on the bronchial epithelium corresponding to the initial lesion site. Two months later, the magenta coloration faded, suggesting pathological normalization of the bronchial epithelium thickening. AFI enabled visualization of tumor progression in the bronchi otherwise completely obstructed by the lesion, potentially offering valuable information to determine bronchial resection lines during surgery.

## Graphical abstract

This is a case report of a patient with stage IIB squamous cell carcinoma (SCC) who underwent neoadjuvant chemoimmunotherapy. Bronchoscopic examination following treatment revealed a complete response (CR) in terms of macroscopic appearance. However, autofluorescence imaging (AFI) still showed a magenta hue at the lesion site, suggestive of residual or altered tissue characteristics. A pathological evaluation was subsequently performed to assess the correspondence between endoscopic imaging and histological findings.

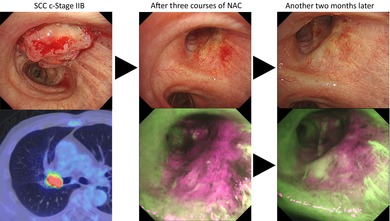

## 1 Introduction

In recent years, indications for immunochemotherapy in lung cancer have expanded from advanced stages to also include early‐stage cases for which surgery is planned [1, 2]. The prognosis for patients achieving a pathological complete response following neoadjuvant immunochemotherapy is excellent [3]. However, more than 10% of patients become ineligible for surgery due to treatment‐related adverse events or disease progression, underscoring that neoadjuvant treatment is a double‐edged sword [2].

Therefore, accurate, image‐based assessment of therapeutic efficacy after neoadjuvant immunochemotherapy is crucial for determining subsequent treatment strategies and predicting patient prognosis. We report a case where treatment response was evaluated in detail using high‐definition bronchoscopy, complemented by novel findings using autofluorescence imaging (AFI).

## 2 Case Report

**FIGURE 1:**
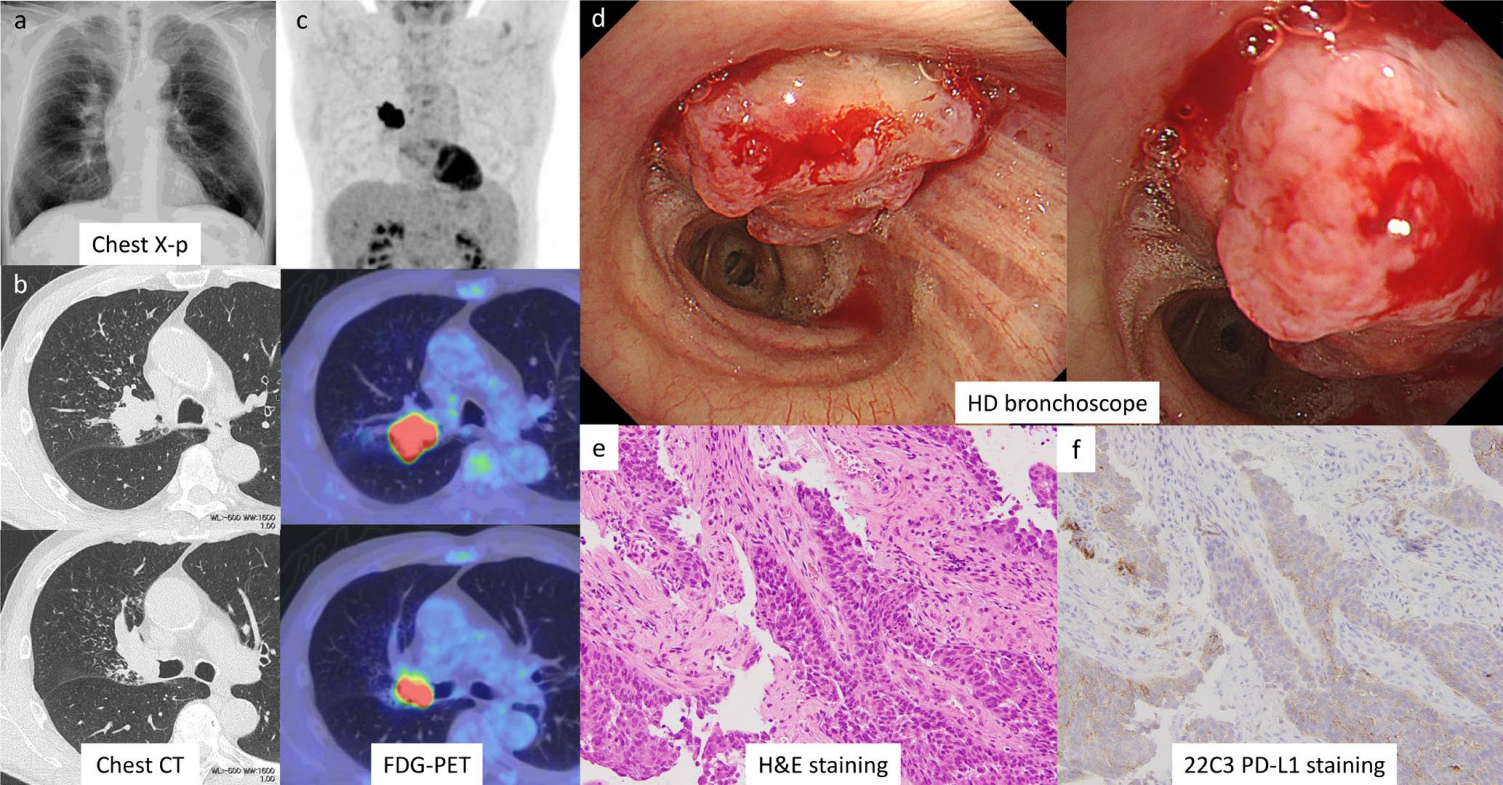
Imaging findings at initial visit, bronchoscopic findings, and histopathological diagnosis. Chest X‐p (a), chest CT (b), and FDG‐PET (c). Tumor obstructing the right upper lobe observed by bronchoscopy (d), and H&E (e) and PD‐L1 (f) staining of biopsy tissue. CT, computed tomography; FDG‐PET, 18F‐fluorodeoxyglucose positron emission tomography; H&E, hematoxylin and eosin, PD‐L1, programmed‐death ligand 1.

A male patient in his 60s with a medical history of alcoholic cirrhosis and type 2 diabetes mellitus presented at a local hospital with a chief complaint of hemoptysis. A chest X‐ray revealed an abnormal shadow, leading to his referral to our hospital (Figure 1a). The patient had an extensive smoking history of 80 cigarettes a day. Chest computed tomography (CT) and fluorodeoxyglucose positron emission tomography (FDG‐PET) scans identified a 43 mm mass located centrally in the right upper lobe (Figure 1b,c). Diagnostic bronchoscopy was subsequently performed. Bronchoscopic diagnosis and observation were performed using the EVIS X1 Video System Center (CV‐1500; Olympus Medical Systems, Tokyo, Japan) in combination with therapeutic bronchoscope: the BF‐1TH1200 for white‐light and NBI imaging, and the BF‐F260 for AFI imaging (both from Olympus Medical Systems, Tokyo, Japan).

Bronchoscopic examination showed complete obstruction of the right upper lobe bronchus by the tumor, preventing visualization beyond the obstruction. Due to the polypoid appearance of the tumor without signs of necrosis (Figure 1d), a direct forceps biopsy was performed. Pathological analysis confirmed squamous cell carcinoma without druggable genetic mutations, and PD‐L1 expression was determined with a tumor proportion score (TPS) of 20%, clinical stage cT2bN1M0 (stage IIB) (Figure 1e,f). The patient underwent neoadjuvant immunochemotherapy with carboplatin, paclitaxel, and nivolumab, administered at 80% standard doses (400, 280, and 240 mg, respectively), with only minor adverse events.

**FIGURE 2:**
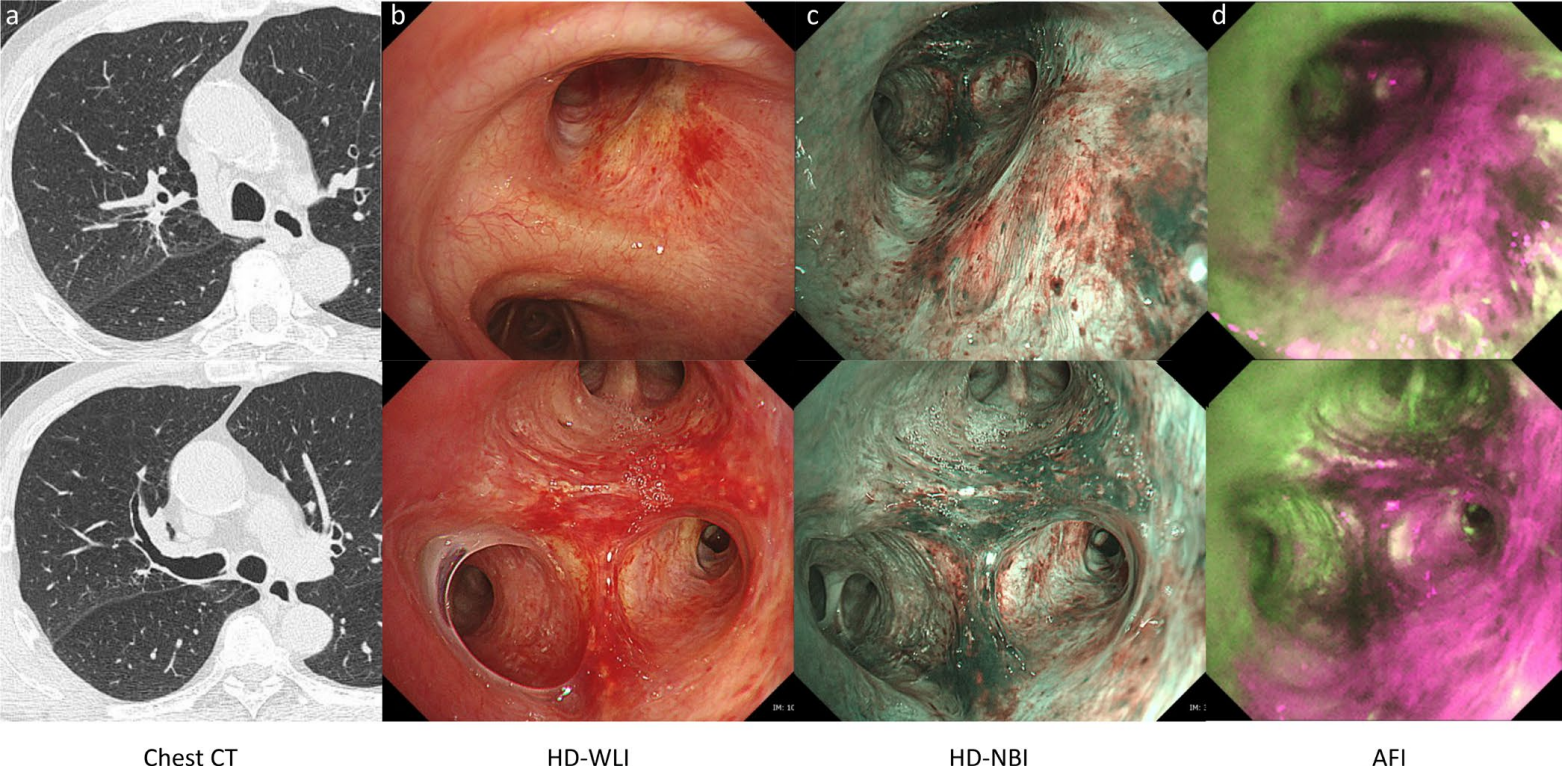
Chest CT (a) and bronchoscopy images immediately after neoadjuvant immunochemotherapy. Bronchoscopy images are in the order of white light (b), NBI (c), and AFI (d). AFI, autofluorescence imaging; CT, computed tomography; HD‐WLI, high definition‐white light imaging; NBI, narrow band imaging.

**FIGURE 3:**
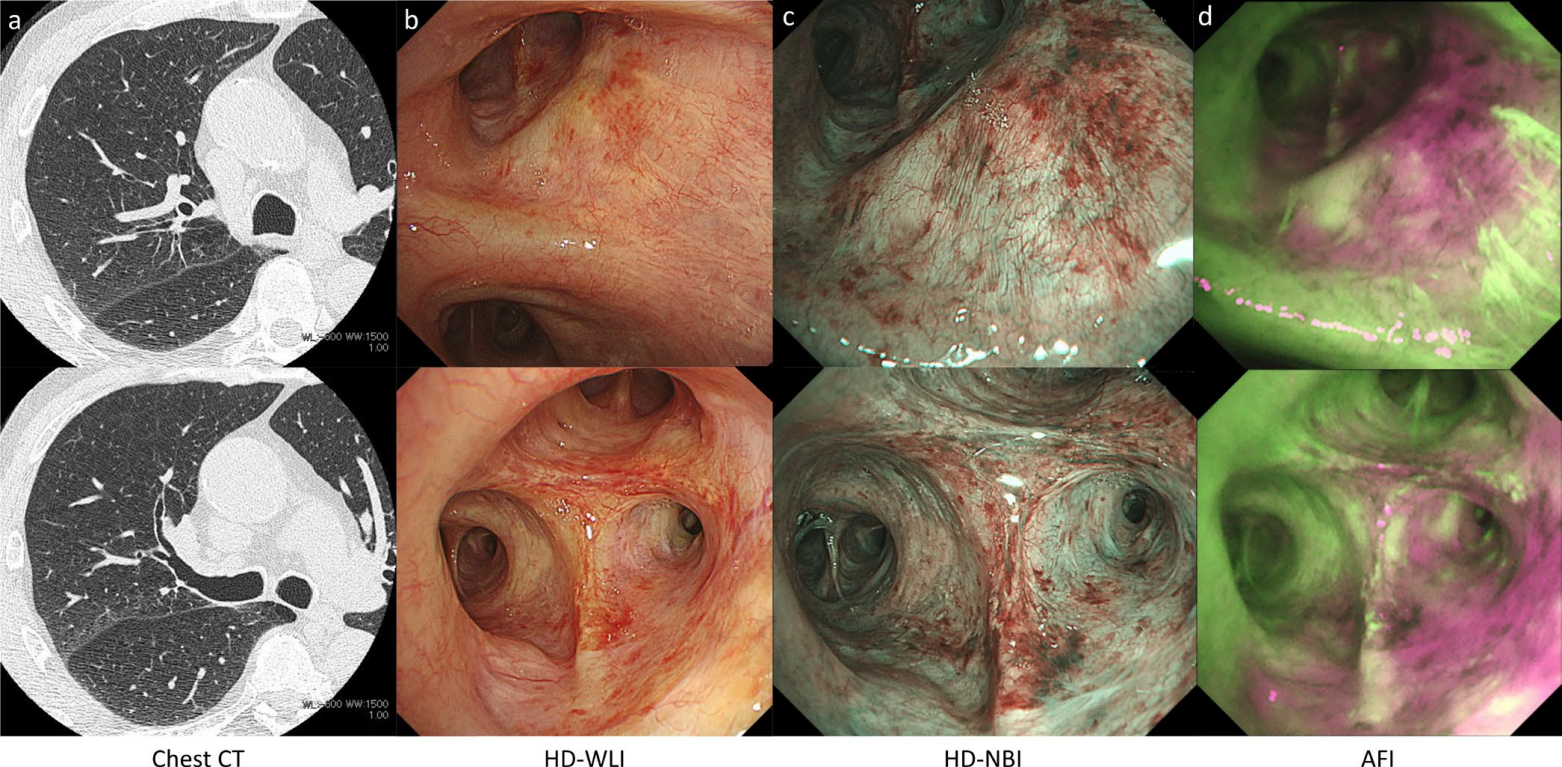
Two and a half months after Figure 2, chest CT (a) and bronchoscopic findings (b–d) immediately before surgery. AFI, autofluorescence imaging; CT, computed tomography; HD‐WLI, high definition‐white light imaging; NBI, narrow band imaging.

After three cycles of neoadjuvant immunochemotherapy, chest CT and bronchoscopy demonstrated complete patency of the right upper lobe bronchus, with no macroscopically visible tumor (Figure 2a,b). Narrow‐band imaging (NBI) showed normal subepithelial vascular patterns without irregularities (Figure 2c). However, AFI highlighted a magenta coloration corresponding to the original tumor site, extending specifically toward the B^2^ bronchus (Figure 2d). Lung resection was postponed by 2 months due to the need for further assessment of esophageal varices. During repeat bronchoscopy and chest CT before surgery, the magenta coloration was observed to have partially resolved, suggesting improvement in the bronchial epithelium thickening (Figure 3a–d).

**FIGURE 4:**
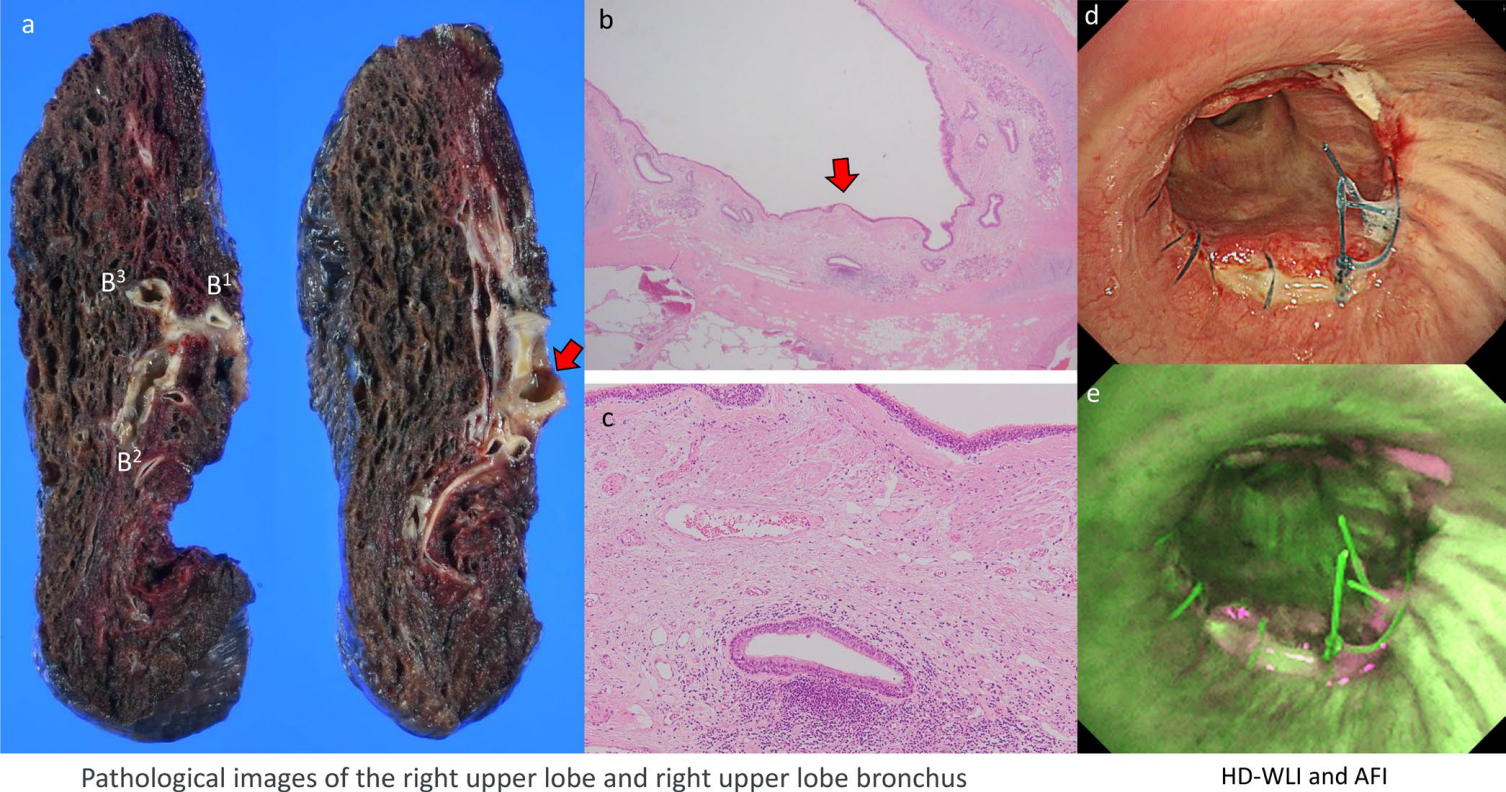
Pathological images of the right upper lobe and right upper lobe bronchus and bronchoscopic images after surgery. The red arrow indicates the right upper lobe bronchus (a, b), and the enlarged pathological image shows extensive fibroelastic change under the epithelium (c). Postoperative bronchoscopy shows white light (d) and AFI (e). AFI, autofluorescence imaging; HD‐WLI, high definition‐white light imaging.

The patient underwent a right upper sleeve lobectomy with bronchoplasty. Pathological analysis confirmed negative bronchial margins, although residual squamous cell carcinoma was detected in the resected lung, within the hilar vessels identified as pulmonary arteries through Elastica van Gieson staining. The TPS of the residual tumor was 0%. While tumor cells were absent from the membranous part of the right upper lobe bronchus, persistent thickening of elastic fibers and infiltration of inflammatory cells were noted, consistent with the bronchoscopic findings of subepithelial thickening (Figure 4a–c). Postoperative bronchoscopic evaluation was performed to confirm findings (Figure 4d,e).

## 3 Discussion

AFI is generally used to assess the extent of epithelial tumor invasion and enhance the sensitivity of detecting early‐stage lung cancer [4, 5, 6, 7, 8, 9, 10, 11], however, there have been limited reports describing its application in other clinical scenarios [12, 13, 14, 15, 16].

In this case, AFI facilitated visualization of tumor progression within bronchial regions that had been completely obstructed by the lesion and were otherwise not observable. After achieving a bronchoscopic complete response to neoadjuvant immunochemotherapy, the original tumor‐contact area, difficult to distinguish by white‐light observation, became clearly visible with AFI as a distinct magenta contrast. Moreover, the most prominent color change was observed in the B^2^ bronchus of the right upper lobe, suggesting that the tumor had proliferated polypoidally from the peripheral B^2^ area—consistent with initial CT findings.

Previous reports indicated that AFI presents magenta coloration due to post‐treatment bronchial epithelium thickening; however, its clinical usefulness has not been clearly established. This case suggests that AFI could be utilized to estimate tumor extent more precisely. Additionally, the observed partial disappearance of magenta coloration over a 2‐month interval implies rapid histological remodeling of the bronchial epithelium. Pathological findings corroborated the bronchoscopic observations, demonstrating persistent thickening of elastic fibers and inflammatory cell infiltration. Although changes in bronchoscopic findings before and after NAC are frequently encountered in daily clinical practice, detailed and multi‐modal imaging evaluations have rarely been reported. On the other hand, the absence of baseline AFI images at the time of initial diagnosis is a limitation of this case.

This case highlights the dramatic reduction in tumor size achievable with neoadjuvant immunochemotherapy and underscores the importance of adequate specimen collection during initial diagnostic evaluation. Traditionally, even with limited initial tumor tissue for biopsy samples, subsequent surgical specimens could yield sufficient material for a definitive morphologic or molecular pathological diagnosis. However, this approach is unsuitable for perioperative treatment cases. If surgical specimens indicate a pathological complete response or major pathological response and no suitable re‐biopsy sites are available during postoperative recurrence, genetic panel testing and TPS assessment must rely on initial diagnostic samples. Therefore, during initial evaluations, it is critical to collect sample specimens, perform essential analyses, and store sufficient tissue samples.

In conclusion, this case illustrates that combining AFI with high‐definition bronchoscopy during neoadjuvant treatment for early‐stage lung cancer allows for the estimation of lesion extent, particularly in bronchial regions previously affected by tumors.

## Author Contributions

K.M. had full access to data presented and takes responsibility for the integrity and accuracy of data analysis. K.M. and K.K. contributed to bronchoscopic examinations and interpretation of findings. K.K., H.M., and H.S. conducted chest surgery. Y.S. and J.K. evaluated pathological findings. K.M., K.K., H.S., and M.M. contributed to scientific review and approved the final manuscript. All authors read and approved the final manuscript.

## Ethics Statement

Written informed consent was obtained from the patient for publication of this case presentation and any accompanying images.

## Conflicts of Interest

The authors declare no conflicts of interest.

## Data Availability

All relevant data are provided within the manuscript. Additional images are available upon request.
